# Self-Healing Performance Assessment of Bacterial-Based Concrete Using Machine Learning Approaches

**DOI:** 10.3390/ma15134436

**Published:** 2022-06-23

**Authors:** Xu Huang, Jessada Sresakoolchai, Xia Qin, Yiu Fan Ho, Sakdirat Kaewunruen

**Affiliations:** 1 Laboratory for Track Engineering and Operations for Future Uncertainties (TOFU Lab), School of Engineering, University of Birmingham, Birmingham B152TT, UK; xxh689@student.bham.ac.uk (X.H.); jss814@student.bham.ac.uk (J.S.); xxq815@student.bham.ac.uk (X.Q.); yxh923@alumni.bham.ac.uk (Y.F.H.); 2 Department of Civil Engineering, School of Engineering, University of Birmingham, Birmingham B152TT, UK

**Keywords:** machine learning-aided prediction, self-healing concrete, bacterial-based self-healing concrete, K-fold cross validation, autonomous healing concrete

## Abstract

Bacterial-based self-healing concrete (BSHC) is a well-known healing technology which has been investigated for a few decades for its excellent crack healing capacity. Nevertheless, considered as costly and time-consuming, the healing performance (HP) of concrete with various types of bacteria can be designed and evaluated only in laboratory environments. Employing machine learning (ML) models for predicting the HP of BSHC is inspired by practical applications using concrete mechanical properties. The HP of BSHC can be predicted to save the time and cost of laboratory tests, bacteria selection and healing mechanisms adoption. In this paper, three types of BSHC, including ureolytic bacterial healing concrete (UBHC), aerobic bacterial healing concrete (ABHC) and nitrifying bacterial healing concrete (NBHC), and ML models with five kinds of algorithms consisting of the support vector regression (SVR), decision tree regression (DTR), deep neural network (DNN), gradient boosting regression (GBR) and random forest (RF) are established. Most importantly, 22 influencing factors are first employed as variables in the ML models to predict the HP of BSHC. A total of 797 sets of BSHC tests available in the open literature between 2000 and 2021 are collected to verify the ML models. The grid search algorithm (GSA) is also utilised for tuning parameters of the algorithms. Moreover, the coefficient of determination (R^2^) and root mean square error (RMSE) are applied to evaluate the prediction ability, including the prediction performance and accuracy of the ML models. The results exhibit that the GBR model has better prediction ability (R^2^_GBR_ = 0.956, RMSE_GBR_ = 6.756%) than other ML models. Finally, the influence of the variables on the HP is investigated by employing the sensitivity analysis in the GBR model.

## 1. Introduction

Considering that concrete has high compressive strength, excellent workability and a low price, and that it can adapt to a vast range of environmental changes effectively, it has been widely used in the construction industry. Crack formation is an ordinary phenomenon in concrete, mainly caused by the ecological influences that lead to low concrete tensile strength. In general, its tensile strength is only 10–15% of its compressive strength [1]. Additionally, temperature changes and extreme weather can also lead to changes in the moisture content and internal drying shrinkage in the concrete. In common sense, small cracks less than 0.2 mm are not considered as a severe case [2]. However, the durability of concrete structures can be dramatically affected by cracks wider than 0.2 mm and, at the same time, internal cracks are not always visible during inspection on a large proportion of concrete structures [3]. Moreover, the manual method of repairing concrete cracks is restricted due to such pessimistic conditions as the environmental impacts and the limited space of operation [4]. The cost of repairing cracked concrete structures accounts for half of the construction budget because of the complex operations, which are considered as another problem [5]. Therefore, to achieve a more effective repairing method and to decrease maintenance funding, an ideal approach should be taken instead of repairing the structure and filling the cracks manually to keep concrete working functionally. A technology named self-healing concrete, which can automatically repair cracks to reduce the maintenance cost and save the environment, was proposed. Self-healing concrete is classified into autogenous healing concrete and agent-based healing concrete [6]. Autogenous healing concrete can be achieved owing to two main important mechanisms: the continuing hydration of un-hydrated cement particles and the carbonation of calcium hydroxide [7]. However, the autogenous healing method has its limits, as it is only useful for tiny cracks less than 300 µm. Concerning the trending agent-based healing, it can help concrete heal itself with various healing agents and is therefore considered as the next-generation technology for concrete. Cracks with widths of up to 970 μm can be repaired employing agent-based healing [8]. The healing agents consist of carriers and core materials with different potentials to heal cracks in concrete. With regard to core materials, bacteria, polymer and expanded materials are employed based on the fact that different healing agents have different healing mechanisms. Thus, BSHC is researched in this paper.

Machine learning is a kind of artificial intelligence. The aim of ML is to obtain the independent prediction ability by learning from input data sets. In this paper, the HP of BSHC is predicted by employing various ML algorithms. Two researchers have studied the HP prediction of BSHC. In their research, the crack closure percentage of non-ureolytic bacterial healing concrete was predicted by employing ML models. Dosages of bacteria, the initial cracking width and the healing time were considered as the inputs of ML models [9]. Moreover, the HP of agent-based healing concrete with a lightweight aggregate (LWA) was predicted by utilising an algorithm combining genetic and ANN algorithms. The initial cracking width, the healing time, the weight of the LWA and the LWA with bacteria were selected as the inputs [10]. It is essential to consider more factors influencing the HP of BSHC due to the complexity of the healing mechanisms. Main influencing factors consisting of the bacteria, the healing environment and the cementitious materials are comprehensively discussed in Section 1.3 of this paper.

In this paper, complete variables (22 influencing factors) are firstly proposed for predicting the HP of BSHC by employing ML models with five types of algorithms. A total of 797 sets of BSHC are collected, and the 22 influencing factors are set as the inputs while the HP is recognised as the unique output. Then, a hyperparameter optimisation method named GSA is utilised to tune the parameters of the five types of ML models. Subsequently, the R^2^ and RMSE, which can indicate the prediction ability of the ML models, are obtained by training the ML models with five algorithms. Then, the optimal ML model for predicting the HP of BSHC is defined according to the R^2^ and RMSE. Moreover, the 10-fold cross validation method is applied to validate the prediction ability of the optimal ML model. Finally, a sensitivity analysis is also conducted on the optimal ML model to investigate the primary influencing variables.

### 1.1. Types of BSHC

Three types of BSHC studied over the past few decades are considered in this paper: UBHC, ABHC and NBHC. Their common healing mechanism is to form calcium carbonate using calcium and carbonate ions generated by various types of bacteria and carbon sources, accordingly. The detailed healing mechanism of each type of BSHC is explained in the following sections.

#### 1.1.1. Ureolytic Bacterial Healing Concrete (UBHC)

UBHC has been studied for a long time because of its fast calcium carbonate production (10 g calcium carbonate production per day) [8]. The healing mechanism of UBHC contains six main steps explained in Equations (1)–(6). The advantage of UBHC is that concrete cracks can be rapidly healed by employing UBHC. However, ammonia and nitrogen oxides generated by UBHC may cause severe damage to the respiration system of creatures. Moreover, unsolidified calcium carbonate coming from too-fast reactions can result in poor performance on strength regain or permeability tests of UBHC [11].

(1) $$\mathrm{CO}{\left({\mathrm{NH}}_{2}\right)}_{2}+H_{2}O\overset{\mathrm{Urease}}{\to }{\;\mathrm{NH}}_{2}\mathrm{COOH}+{\mathrm{NH}}_{3}\;$$

(2) $${\mathrm{NH}}_{2}\mathrm{COOH}+H_{2}O\to {\mathrm{NH}}_{3}+H_{2}{\mathrm{CO}}_{3}\;$$

(3) $$2{\mathrm{OH}}^{-}+H_{2}{\mathrm{CO}}_{3}\to {\mathrm{CO}}_{3}^{2-}+2H_{2}O$$

(4) $$2{\mathrm{NH}}_{3}+2H_{2}O\to 2{\mathrm{NH}}_{4}^{+}+2{\mathrm{OH}}^{-}$$

(5) $$\mathrm{CO}{\left({\mathrm{NH}}_{2}\right)}_{2}+2H_{2}O\to 2{\mathrm{NH}}_{4}^{+}+{\mathrm{CO}}_{3}^{2-}\;$$

(6) $${\mathrm{CO}}_{3}^{2-}+{\mathrm{Ca}}^{2+}\to {\mathrm{CaCO}}_{3}↓$$

#### 1.1.2. Aerobic Bacterial Healing Concrete (ABHC)

The healing mechanism of ABHC is to heal cracks using calcium carbonate produced by the aerobic metabolism conversion of organic acids employing alkali-resistant bacteria such as *Bacillus cohnii*. Organic acids, such as calcium lactate and calcium formate, are recognised as carbon sources to provide carbonate ions, which can react with existing calcium ions to produce calcium carbonate [12]. This healing method is exhibited in Equations (7) and (8) [13].

(7) $${\mathrm{CaC}}_{6}H_{10}O_{6}+6O_{2}\to {\mathrm{CaCO}}_{3}+5{\mathrm{CO}}_{2}+5H_{2}O$$

(8) $$5{\mathrm{CO}}_{2}+5\mathrm{Ca}{\left(\mathrm{OH}\right)}_{2}\to 5{\mathrm{CaCO}}_{3}+5H_{2}O$$

#### 1.1.3. Nitrifying Bacterial Healing Concrete (NBHC)

The healing mechanism of NBHC is different from that of UBHC and ABHC. Oxygen is essential to UBHC and ABHC. However, cracks can be healed under the oxygen-limited condition by employing NBHC [14]. The healing mechanism of NBHC involves nitrate ions being reduced to nitrite ions by the reaction with organic carbon such as formate [15,16]. The healing mechanism of NBHC can be explained as follows in Equations (9)–(11). The main drawback of NBHC is that it is costly to cultivate bacteria in the oxygen-free and axenic environment [16].

(9) $$2{\mathrm{NO}}_{3}^{-}+2{\mathrm{HCOO}}^{-}+2H^{+}\to 2{\mathrm{CO}}_{2}+2{\mathrm{NO}}_{2}^{-}+2H_{2}O$$

(10) $${\mathrm{Ca}}^{2+}+{\mathrm{CO}}_{2}+H_{2}O\to 2H^{+}+{\mathrm{CaCO}}_{3}↓\;$$

(11) $$2{\mathrm{NO}}_{2}^{-}+3{\mathrm{HCOO}}^{-}+5H^{+}\to 3O_{2}+N_{2}+4H_{2}O\;$$

According to the detailed healing mechanism explanation, it can be observed that different types of BSHC require different kinds of bacteria, nutrients, healing environments, etc. Moreover, different HPs can be achieved when different types of BSHC are employed.

### 1.2. Types of Bacteria

**Figure 1:**
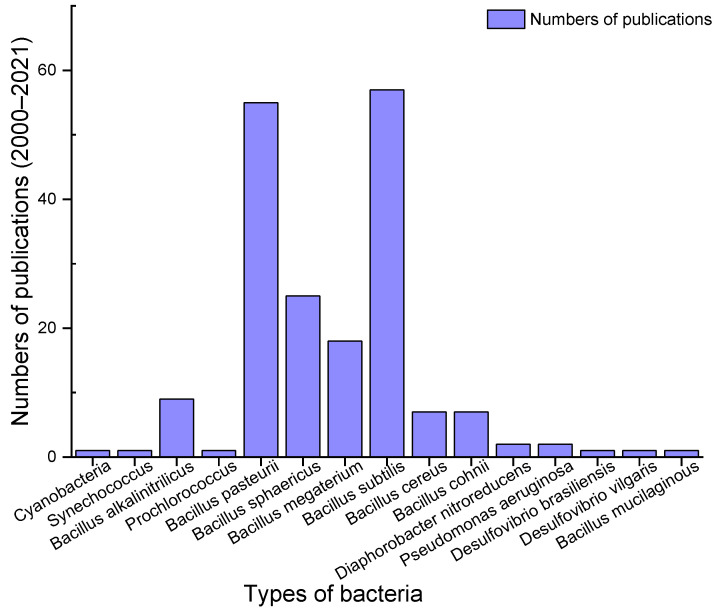
Numbers of publications of bacteria related to BSHC.

Published articles related to BSHC between 2000 and 2021 are collected and analysed in this paper. According to the record, 15 types of bacteria have been employed for BSHC experiments as shown in Figure 1. Thereinto, *Cyanobacteria*, *Synechococcus*, *Prochlorococcus Bacillus alkalinitrilicus*, *Bacillus subtilis*, *Bacillus cohnii*, *Pseudomonas aeruginosa* and *Bacillus mucilaginous* belong to ABHC. *Bacillus pasteurii*, *Bacillus sphaericus*, *Bacillus megaterium* and *Diaphorobacter nitroreducens* can be classified into NBHC. *Bacillus cereus*, *Desulfovibrio brasiliensis* and *Desulfovibrio vulgaris* can be concluded as UBHC and sulphate reduction biological mineralisation, respectively. It can be observed from Figure 1 that seven types of bacteria, i.e., *Bacillus pasteurii*, *Bacillus sphaericus*, *Bacillus megaterium*, *Bacillus subtilis*, *Bacillus cereus*, *Bacillus alkalinitrilicus* and *Bacillus cohnii*, have been employed more commonly. The rest of the bacteria, such as *Cyanobacteria* and *Pseudomonas aeruginosa*, have been utilised less than twice. Therefore, only the experimental data containing these seven types of bacteria are collected in this paper. Then, the experimental data consisting of all the 22 variables are utilised and input into the ML models in this paper.

### 1.3. Influencing Factors of HP

The HP of BSHC is dependent on complicated processes, including physical and chemical reactions. Three main aspects of influencing factors are considered in this paper. Firstly, influencing factors related to bacteria, such as types of bacteria, dosages of bacteria, types of nutrients and types of carriers, are investigated [12,17,18]. Secondly, types of cement and water binder ratios are considered as the influencing factors related to cementitious materials and water contents. Thirdly, healing conditions, the initial cracking width and the initial cracking date are influencing factors associated with the healing environment [7,18,19,20,21,22]. Therefore, 22 influencing factors regarding the three aspects are considered as variables of the ML models in this paper. The detailed description of the 22 influencing factors can be found in Section 2.1.

### 1.4. Healing Performance Determination

In order to investigate the healing efficiency of BSHC, the HP is introduced. HP represents the percentages of cracks that can be repaired, and it can be calculated by Equation (12) based on the initial cracking condition and the final cracking condition measurement [18]. The cracking conditions are evaluated by five types of measurement methods, i.e., the cracking width measurement, the cracking area measurement, the ultrasound pulse velocity measurement, the regained strength measurement and the anti-seepage repairing measurement.

(12) $$HP=\frac{c_{w_{i}}-c_{w_{t}}}{c_{w_{i}}}\times 100.$$

where $c_{w_{i}}$ is the initial cracking condition, $c_{w_{t}}$ is the final cracking condition measured in specific curing time and *HP* is the healing performance.

## 2. Materials and Methods

### 2.1. Data Preparation

A total of 797 data sets employed for predicting the HP of BSHC were collected from 14 articles published between 2000 and 2021 [18,23,24,25,26,27,28,29,30,31,32,33,34,35]. As mentioned in Section 1.3, 22 variables influencing the HP of BSHC are employed in this paper to train ML models with the five algorithms. Six variables are used to describe the influencing factors of cementitious materials and water contents: the amount of fine aggregate (FA), the amount of coarse aggregate (CA), types of cement (TC), the amount of cement (CM), the water binder ratio (W/B) and the amount of superplasticiser (S). Furthermore, the eleven variables corelated with bacteria are the types of carriers (C), types of bacteria (B), dosages of bacteria (DB), types of BSHC (TBSHC), types of calcium ions sources (TCIS), dosages of calcium ions (DCI), types of carbon sources (TCS), dosages of carbon (DC), types of nutrients (TN), dosages of nutrients (DN) and dosages of urea (DU). All variables are represented by the mass ratio of concrete. Moreover, the initial cracking date (CD), the initial cracking width (CW), the healing time (HT), the healing condition (HC) and the healing test methods (HTM) are the variables with reference to the healing environment. Finally, the self-healing efficiency is represented by the healing performance (HP) as the unique output. Table 1 exhibits the ranges of the 22 variables. Variables such as the types of bacteria and carriers are replaced by numeric values, explained in Table A1, Table A2, Table A3, Table A4, Table A5, Table A6, Table A7, Table A8 and Table A9 in Appendix A. After the data preparation, the collected data sets utilise between zero and one by the following calculation in Equation (13) [36].

(13) $$\mathrm{Normalised}\;\mathrm{value}=\frac{x-x_{\min}}{x_{\max}-x_{\min}}$$

where x is the data value and $x_{\min}$ and $x_{\max}$ are the minimum and maximum values, respectively.

### 2.2. Machine Learning Algorithms

Five types of ML algorithms, GBR, RF, DNN, DTR and SVR, have been extensively developed for predicting the mechanical properties of concrete utilising empirical data [37,38]. For instance, ANN and MLR models were employed to predict the 28-day compressive strength of concrete. The ANN model obtained an R^2^ value of 0.9226, which was dramatically higher than that of the MLR model (R^2^_MLR_ = 0.7456). Furthermore, R^2^ values of 0.951 and 0.929 for predicting the compressive and splitting tensile strength were demonstrated by employing GBR models [24,39,40,41,42,43,44,45,46,47,48,49,50,51]. In this paper, the prediction ability of the five types of ML models for predicting the HP of BSHC is studied. To achieve the best prediction ability, here a hyper-parameter tuning method named GSA is utilised to determine the optimal parameters of the ML models [52]. The reason why GSA is a reliable hyper-parameter tuning method can be attributed to its ability to find the optimal hyper-parameters combination according to an exhaustive analysis [53,54,55,56].

### 2.3. Prediction Ability Evaluation

The prediction ability of the ML models with five algorithms for predicting the HP of BSHC is evaluated by the coefficient of determination ($R^{2}$) and the root mean square error (RMSE). The RMSE is the arithmetic root of mean square error (MSE) and is also called the standard error. The RMSE is sensitive to the extreme errors of prediction values. Therefore, the prediction accuracy can be precisely reflected by the RMSE. RMSE values are calculated according to Equation (14). A lower RMSE exhibits a higher accuracy of ML models [37]. Moreover, the $R^{2}$ is a significant statistical magnitude to evaluate the prediction performance of ML models ranging from zero to one. A higher value of $R^{2}$ means a better performance of ML models [57]. Equation (15) explains the $R^{2}$.

(14) $$\;\mathrm{RMSE}=\sqrt{\frac{\sum_{i=1}^{n}{\left(y_{i}^{\prime }-y_{i}\right)}^{2}}{n}}$$

(15) $$R^{2}=1-\frac{\sum_{i=1}^{n}{\left(y_{i}^{\prime }-y_{i}\right)}^{2}}{\sum_{i=1}^{n}{\left(y_{i}\;-\;\overset{\text{¯}}{y}\right)}^{2}}$$

where $\left(y_{i}^{\prime }-y_{i}\right)$ indicates the difference between real and predicted values and n stands for the number of measurements.

### 2.4. Data Splitting

The data sets employed in this paper are randomly split into the training and testing sets with a ratio of 8:2, respectively. The data in the training set (80%) are applied to tune the ML models. Moreover, the data in the testing set (20%) are employed to inspect the generalisation capacity of the ML models, i.e., the testing data set is recognised as a new data set to fit the ML models after conducting the training process.

## 3. Results

### Prediction Ability of ML Models

**Figure 2:**
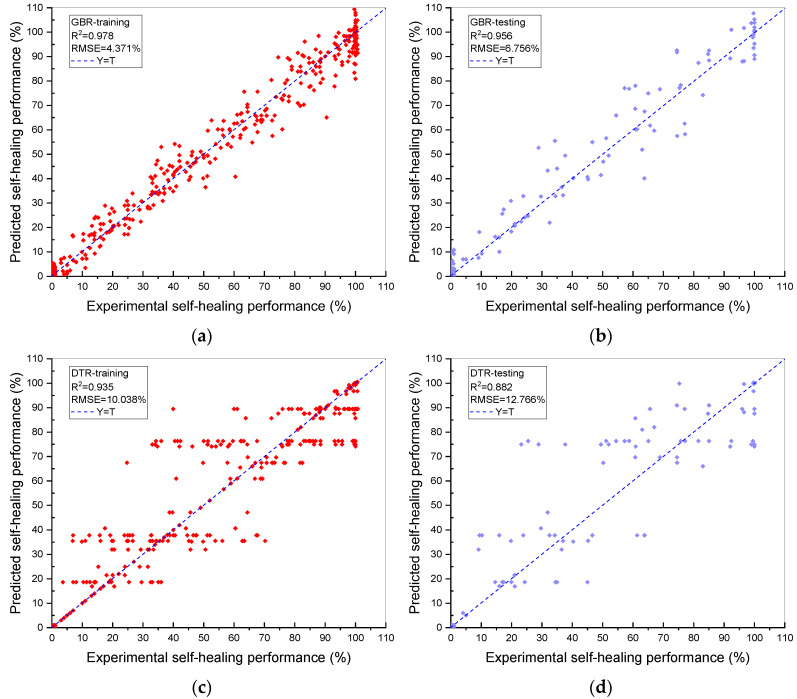

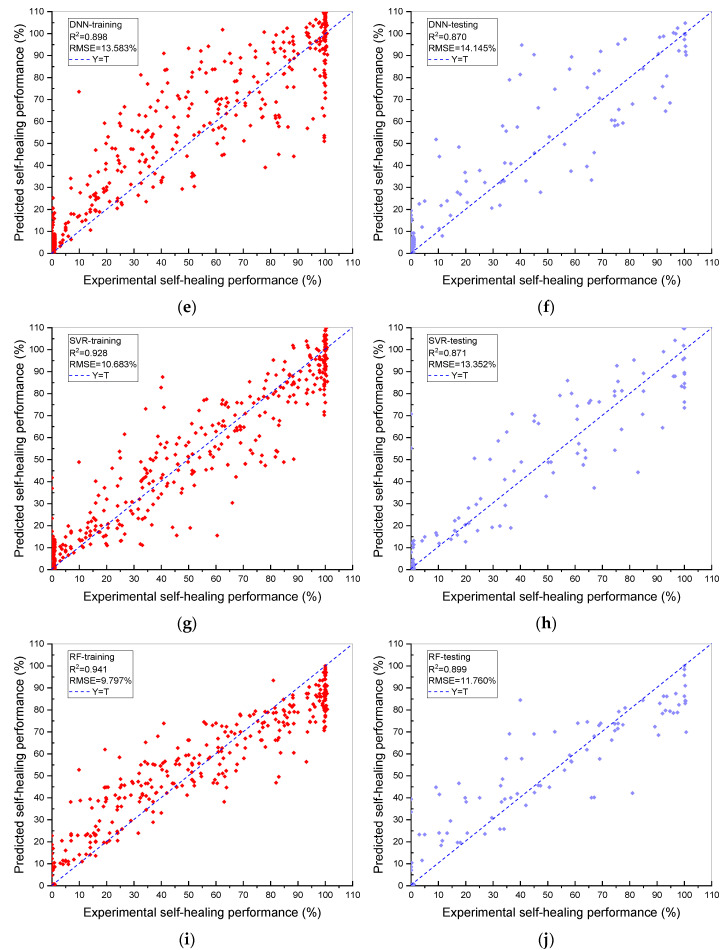
Experimental vs. predicted HP for the models: (**a**) GBR-training; (**b**) GBR-testing; (**c**) DTR-training; (**d**) DTR-testing; (**e**) DNN-training; (**f**) DNN-testing; (**g**) SVR-training; (**h**) SVR-testing; (**i**) RF-training; and (**j**) RF-testing, with the corresponding R^2^ and RMSE.

The prediction ability (R^2^ and RMSE values) of the training and testing data sets by the five types of ML models demonstrating the relationship between the predicted and experimental HP of BSHC is exhibited in Figure 2. R^2^ and RMSE values are applied to inspect the prediction performance and accuracy of the ML models. The horizontal and vertical axes indicate the experimental and predicted HP, respectively. Furthermore, the results of the ML models are demonstrated in Table 2 to show the differences in the prediction ability. Moreover, the optimal parameters of the ML models defined by GSA are listed in Table 3.

As is demonstrated in Figure 2a,b, the GBR model shows a significantly higher R^2^ than the other four ML models. The R^2^ and RMSE of GBR are 0.956 and 6.756%, respectively. Furthermore, the R^2^ and RMSE values of DNN, DTR, RF and SVR are (0.870, 14.145%), (0.882, 12.766%), (0.899, 11.760%) and (0.871, 13.352%), respectively, which are lower than that of the GBR model (Figure 2c,j). According to the results, the following can be concluded. Firstly, the GBR model is the optimal model for predicting the HP of BSHC due to the highest R^2^ (0.956) and lowest RMSE (6.756%). Secondly, the GBR model is reliable because of the similar R^2^ results of the training and testing sets, indicating no underfitting or overfitting problem. Thirdly, the RMSE (6.756%) of the GBR model demonstrates that the prediction deviation is low and robust.

## 4. Discussion

The optimal ML model for predicting the relationship between the 22 variables and the HP of BSHC, GBR, is defined by the best prediction ability results and slight differences between the experimental and predicted HP shown in Figure 2. The reason why the GBR model has a better prediction ability than the other models can be concluded as follows. ML models with the GBR algorithm, named as ensemble ML models, have an excellent regression capacity and an extraordinary generalisation ability due to the applied boosting strategy. Different weights are distributed to weak learners generated by the boosting strategy in accordance with the prediction ability of weak learners. Namely, weak learners with a better prediction ability can obtain higher weights. The promising prediction ability of GBR models can be investigated when a strong learner is composed of all weak learners, while the other ML models have a lower prediction ability because they are individual algorithms [58].

### 4.1. K-Fold Cross Validation

K-fold cross validation is a method to validate the prediction ability of the optimal ML model, GBR. In this paper, the prediction ability of GBR is validated by employing 10-fold cross validation. The 10-fold cross validation method can be described as follows. Firstly, 797 data sets are divided into 10 sections. Then, some data sets are employed to train GBR models, while the rest of the data sets are utilised to validate the trained GBR models. Subsequently, the first step is conducted ten times with different training and testing data set groups. Finally, the prediction ability of the GBR model validated by 10-fold cross validation can be generated by means of averaging the R^2^ and RMSE values of all GBR models [59].

**Figure 3:**
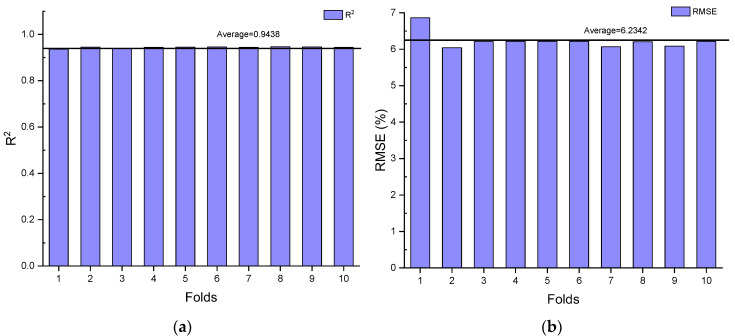
(**a**) R^2^ results and (**b**) RMSE results of GBR models with the 10-fold cross validation for predicting HP of BSHC.

The prediction ability (R^2^ and RMSE values) of the GBR models validated by different folds of the data sets is shown in Figure 3. Slight differences in R^2^ and RMSE values of the GBR models can be noticed in Figure 3a,b. For instance, 0.947 is the maximum R^2^ value of the GBR model at Fold 8, while 0.937 is the minimum R^2^ value of the GBR model at Fold 1. The rest of the R^2^ values are maintained at approximately 0.944. Furthermore, the RMSE value dramatically decreases from 6.864% to 6.039% between Fold 1 and Fold 2, followed by a slight growth to 6.210% at Fold 3. Subsequently, it keeps constant at 6.218% until Fold 6. It then fluctuates between 6.067% and 6.218% from Fold 7 to Fold 10. Moreover, the average R^2^ and RMSE values and the standard deviations (SDs) of the GBR models are listed in Table 4. The average R^2^ and RMSE values of the GBR models with different folds of the data sets are 0.9438 and 6.2342%, respectively. Additionally, the SDs of the R^2^ and RMSE values are 0.0029 and 0.2208, respectively, which can be concluded that the coefficient of variations (COVs) of the values are relatively low, only 0.31% and 3.54%, respectively. Regarding the R^2^, RMSE and the statistical results of the GBR models, it can be concluded that the promising prediction ability of the GBR model for predicting the HP of BSHC is reliable.

### 4.2. Sensitivity Analysis

Sensitivity analysis (SA) is a type of machine learning interpretation. Moreover, it is an uncertainty analysis method to study the influence of variables on the output from quantitative analysis. In this paper, the optimal ML model for predicting the HP of BSHC, GBR, is employed for SA. The main processes of SA can be defined as follows. Firstly, the values of one variable are kept consistent with the collected experimental data at a time, while the rest of the variables are kept constant at the mean values. Subsequently, the new data sets are applied to train the optimal ML model, GBR. Finally, Equation (16) is employed to investigate the corresponding sensitivity analysis parameter (SAP) of each variable.

(16) $$SAP_{i}=\frac{HP_{max}\left(V_{i}\right)-HP_{min}\left(V_{i}\right)}{\sum_{i}\left[HP_{max}\left(V_{i}\right)-HP_{min}\left(V_{i}\right)\right]}\times 100\;$$

where $SAP_{i}$ indicates the SAP of the variable *i* and $HP_{max}\left(V_{i}\right)$ and $HP_{min}\left(V_{i}\right)$ are the maximum and minimum *HP* of the variable *i*.

**Figure 4:**
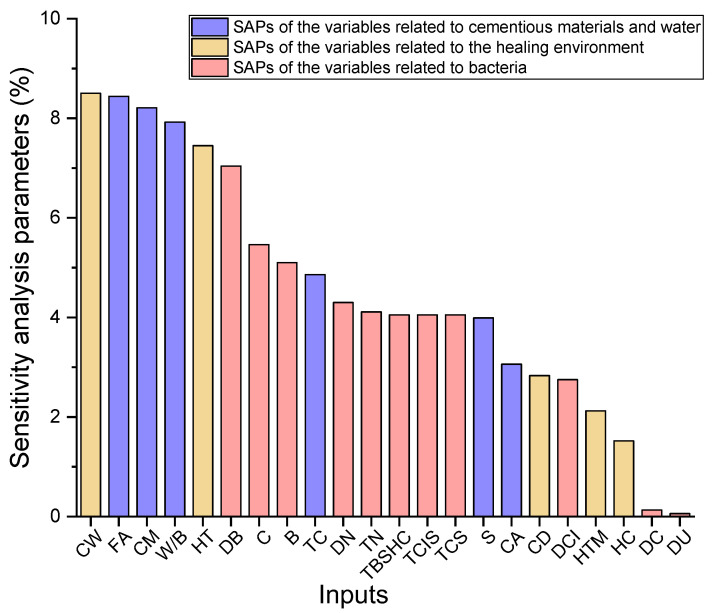
SAPs of the variables.

The SAPs of the variables related to cementitious materials and water, the healing environment and bacteria are shown in Figure 4. The maximum SAP is 8.50% of CW, while the minimum SAP is 0.06% of DU. It can be interpreted that CW has a pronounced influence on the HP of BSHC. However, little effect of urea on the HP of BSHC is observed. The SAPs of FA, CM, W/B, HT and DB are 8.44%, 8.21%, 7.92%, 7.45% and 7.04%, respectively, which are slightly lower than that of CW. Subsequently, the SAP dramatically decreases from 7.04% to 5.46% between DB and C. Then, the SAP experiences a gradual drop from 5.10% to 3.99% between B and S. The SAPs of C, TC, DN, TN, TBSHC, TCIS and TCS are 5.10%, 4.86%, 4.30%, 4.11%, 4.05%, 4.05% and 4.05%, respectively. Additionally, the rest of the variables demonstrate a relatively lower influence on HP, i.e., 3.06%, 2.83%, 2.75%, 2.12%, 1.52% and 0.13% for CA, CD, DCI, HTM, HC and DC, respectively. With regard to the SAP results, the following aspects can be concluded. Firstly, most of the variables related to cementitious materials and water, such as FA, CM and W/B, show a stronger influence on the HP of BHSC than that of the variables related to bacteria. This is because less water contained in the concrete results in more unreacted cement particles being retained for healing cracks. Furthermore, more FA can lead to the increased demand of water; thus, the HP of concrete with high FA is lower than that of concrete with low FA. It can be concluded that the influence of the variables on the HP of BSHC is CW ≥ water contents > HT > the variables related to bacteria. Secondly, the variables related to the healing environment, such as CW, HT and CD, were recognised as the significant influencing factors of HP [9,10]. However, there was no report to show the influencing degrees of the factors. In this paper, it can be observed from Figure 4 that the SAP of HT is 7.45%, 12.35% lower than that of CW. Moreover, the SAP of CW is more than three times that of CD. Thirdly, regarding the variables related to bacteria, DB has a higher effect than other variables on the HP of BSHC. The SAPs of the rest of the variables related to bacteria are close, excluding DC and DU, indicating a similar influence on the HP of BSHC. It can be observed from Figure 4 that DC and DU show little influence on the HP of BSHC.

## 5. Conclusions

In this paper, five types of ML models for predicting the HP of BSHC were proposed to aid in self-healing concrete design. The ML models were used for the non-linear relationship modelling between HP and its 22 variables, and GSA as the optimal method was applied for the hyper-parameter tuning. A total of 797 data sets were collected through extensive experiments with different combinations of variables for training the ML models.

On the basis of the results, the following conclusions can be drawn:

- The $R^{2}$ and RMSE values of the GBR model were 0.956 and 6.756%, respectively, which means that the prediction performance is excellent, and the prediction deviation is relatively low and reasonable. The GBR model was also compared to other ML algorithms, such as DTR, SVR, DNN and RF, and it showed an outstanding superiority to these ML models. Thus, it can be concluded that GBR is the optimal ML model that can accurately predict the HP of BSHC with the 22 variables.
- Concerning the results of the 10-fold cross validation, the average R^2^ and RMSE values were 0.9438 and 6.2342%, respectively. Thus, it can be concluded that the robust prediction ability of the GBR model is convincing.
- All variables in the GBR model were studied to inspect the influence on the HP of BSHC. It was observed that CW, FA, CM, W/B, HT and DB are key variables and have relatively higher effects on the HP of BHSC, which means that they cannot be neglected during the ML-aided self-healing concrete design.

The HP of BSHC consisting of various variables can be effectively predicted by employing the GBR model in this paper. As a consequence, the GBR model can be utilised to validate the BSHC design and whether its expected HP can be achieved according to the following steps. Firstly, a GBR model should be developed according to the parameters given in Table 3. Secondly, nine types of parameters, such as C, should be replaced by numeric values according to Table A1, Table A2, Table A3, Table A4, Table A5, Table A6, Table A7, Table A8 and Table A9 in Appendix A. Subsequently, the rest of the parameters, such as DB, need to be defined according to the BSHC design. Finally, the GBR model is able to predict the HP of the designed BSHC. Moreover, the BSHC design optimisation can be realised using the GBR model.

## Figures and Tables

**Table 1:** The ranges of the variables (inputs) and the output.

Types of Variables	Symbol	Unit	Minimum	Maximum
Inputs	C	-	0	8
	TC	-	1	3
	B	-	0	6
	DB	cells/g	0	2.6 × 10^9^
	TBSHC	-	0	2
	TCIS	-	1	3
	DCI	g/g	0	0.034
	TCS	-	0	2
	DC	g/g	0	0.034
	TN	-	1	3
	DN	g/L	0	4
	DU	g/L	0	0.024
	FA	g/g	0.204	0.666
	CA	g/g	0	0.522
	CM	g/g	0.156	0.222
	W/B	-	0.4	0.599
	S	g/g	0	1.564
	CD	days	3	56
	CW	mm	0.027	1.152
	HC	-	1	3
	HT	days	3	100
	HTM	-	1	5
Output	HP	%	0	100.76

**Table 2:** R^2^ and RMSE values of the ML models.

Algorithm	Dataset	HP Prediction Ability	
		R^2^	RMSE (%)
GBR	Training	0.978	4.371
	Testing	0.956	6.756
DTR	Training	0.935	10.038
	Testing	0.882	12.766
DNN	Training	0.898	13.583
	Testing	0.870	14.145
SVR	Training	0.928	10.683
	Testing	0.871	13.352
RF	Training	0.941	9.797
	Testing	0.899	11.760

**Table 3:** Tuned parameters of the ML models employing GSA.

Algorithms	Parameters	Setting
DNN	Hidden layers	4
	Hidden neurons	30-30-30-30
	Learning rate	0.0010
	Activation function	Maxout
GBR	Depth_max_	21
	Split_min_	0.001
	Learning rate	0.9001
	Number of trees	21
DTR	Depth_max_	10
	Split_min_	1.000
	Leaf_min_	1
	Gain_min_	0.0010
SVR	C_penalty_	1
	Epsilon	0.001
	Gamma	5000
	Kernel type	Radial
RF	Depth_max_	60
	Split_min_	100.000
	Leaf_min_	60
	Gain_min_	0.3007
	Number of trees	11

**Table 4:** R^2^ and RMSE results of GBR models with the 10-fold cross validation.

Folds	HP Prediction Ability	
	R^2^	RMSE (%)
Fold 1	0.937	6.864
Fold 2	0.945	6.039
Fold 3	0.940	6.210
Fold 4	0.944	6.218
Fold 5	0.945	6.218
Fold 6	0.946	6.218
Fold 7	0.944	6.067
Fold 8	0.947	6.206
Fold 9	0.946	6.084
Fold 10	0.944	6.218
Average	0.9438	6.2342
SD	0.0029	0.2208

## Data Availability

Data can be made available upon reasonable request.

## Appendix A

**Table A1:** Types of carriers.

Number	Representation
0	No carrier
1	Expanded clay
2	Expanded perlite
3	Graphene nanoplatelets
4	Coir
5	Flax
6	Jute
7	Low alkali calcium sulphoaluminate
8	Recycled brick aggregate

**Table A2:** Types of bacteria.

Number	Representation
0	No bacteria
1	*Bacillus subtilis*
2	*Bacillus cohnii*
3	*Bacillus alkalinitrilicus*
4	*Bacillus pasteurii*
5	*Bacillus sphaericus*
6	*Bacillus megaterium*

**Table A3:** Types of healing conditions.

Number	Representation
1	Ambient water condition
2	Ambient air condition
3	Wet–dry cycles

**Table A4:** Types of BSHC.

Number	Representation
0	Autogenous healing
1	ABHC
2	UBHC

**Table A5:** Types of nutrients.

Number	Representation
1	Peptone
2	Yeast
3	Beef extract

**Table A6:** Types of carbon sources.

Number	Representation
0	Water
1	Air
2	Calcium lactate

**Table A7:** Types of cement.

Number	Representation
1	CEM I 42.5N
2	CEM II 42.5N
3	CEM I 52.5N

**Table A8:** Types of calcium ion sources.

Number	Representation
1	Calcium nitrate
2	Calcium lactate
3	Ca(OH)_2_

**Table A9:** Types of healing test methods.

Number	Representation
1	Cracking width measurement
2	Cracking area measurement
3	Ultrasound pulse velocity measurement
4	Regained strength measurement
5	Anti-seepage repairing measurement
